# Contagious yawning in virtual reality is affected by actual, but not simulated, social presence

**DOI:** 10.1038/s41598-018-36570-2

**Published:** 2019-01-22

**Authors:** Andrew C. Gallup, Daniil Vasilyev, Nicola Anderson, Alan Kingstone

**Affiliations:** 1 Psychology Program, SUNY Polytechnic Institute, Utica, NY 13502 USA; 2 Department of Psychology, University of British Columbia, Vancouver, BC V6T 1Z Canada

## Abstract

Contagious yawning occurs in humans and a few other highly social animals following the detection of yawns in others, yet the factors influencing the propagation of this response remain largely unknown. Stemming from earlier laboratory research, we conducted five experiments to investigate the effects of social presence on contagious yawning in virtual reality (VR). We show that, similar to a traditional laboratory setting, having a researcher present during testing significantly inhibited contagious yawning in VR, even though participants were viewing a virtual environment and unable to see the researcher. Unlike previous research, however, manipulating the social presence in VR (i.e., embedding recording devices and humanoid avatars within the simulation) did not affect contagious yawning. These experiments provide further evidence that social presence is a powerful deterrent of yawning in humans, which warrants further investigation. More generally, these findings also have important applications for the use of VR in psychological research. While participants were quite sensitive to social stimuli presented in VR, as evidenced by contagious yawning, our results suggest a major difference in the influence of social factors within real-world and virtual environments. That is, social cues in actual reality appear to dominate and supersede those in VR.

## Introduction

Yawning is characterized by a powerful gaping of the jaw with deep inspiration, followed by a temporary period of peak muscle contraction and a passive closure of the jaw during expiration^1^. Physiologically, yawns enhance intracranial circulation^2^ and facilitate brain cooling^3–5^, which in turn could serve to promote cortical arousal^6^ and state change^7^ during behavioral transitions. Contagious yawning, which represents the reflexive triggering or release of this response as a result of sensing yawns in others, is a well-documented phenomenon in humans, as seeing, hearing and even thinking about yawning can induce yawn contagion^7,8^. Evidence for contagious yawning is also present in a small number of non-human animals, including some non-human primates, birds and domesticated dogs^9^.

Spontaneous and contagious yawns represent intrinsically connected, yet distinct behaviors. Although indistinguishable in the motor action pattern described above, a number of important factors, in addition to their triggers (i.e., physiological vs. social), differentiate these two types of yawns. Spontaneous yawns, or similar mandibular-gaping patterns, appear to be a phylogenetically old and conserved across vertebrate classes^10^, while contagious yawning is a more recently derived feature present in only a few highly social species^11,12^. Consistent with this view, these two responses show distinct ontogenies, with spontaneous yawns emerging early on within intrauterine development in humans^13^, while contagious yawns do not appear until early childhood^14^. Furthermore, spontaneous yawns seem to be a universal act across members of a given species, whereas the expression of yawn contagion appears to show much more individual variability. For example, depending upon the methods and stimuli used, only ~30–60% of people show contagious yawning across laboratory and semi-naturalistic settings^7,8,15–17^, with similarly variable response rates observed for non-human primates^18^.

The fundamental differences between these two yawn-types have led some researchers to propose that yawn contagion may reflect a form of higher-level social-cognitive processing (e.g., emotional contagion or state matching)^19–21^. Indeed, a large body of research has explored the proposed connection between yawning and empathy, with lines of supporting evidence coming from correlational studies^15^, in-group/out-group comparisons^22^, neuroimaging investigations^23^, and clinical studies^24^. Consistent with this view, one study even showed a negative association between contagious yawning and measures of psychopathy in a non-clinical population^25^. However, a recent review of this literature concluded that the empathy/contagious yawning link is weak and inconsistent, with the majority of studies failing to observe the predicted effects derived from this empathy modeling hypothesis^9^. In fact, recent experiments have shown that the susceptibility to contagious yawning among humans is tied to the perceptual encoding of the act in others and is unrelated to psychological traits linked with empathy or emotional processing^26^. Others have argued that it is not necessary to implicate a link between contagious yawning and higher-order cognitive processes^27,28^. For example, given the physiological consequences of this motor action pattern (i.e., enhanced intracranial circulation and brain cooling), the spreading of this response through contagion could have evolved to coordinate group arousal or activity patterns and promote collective vigilance^16,29^. Further research in these areas is needed, but the fact that individual differences in contagious yawning may predict important aspects of psychological and perceptional functioning deserves further attention.

Despite the involuntary and impromptu nature of yawns, and the potential benefits from this action, the expression of yawning is often stigmatized in social settings. In fact, yawns are interpreted as a sign of boredom and disrespect across different cultures^30^. This seemingly widespread, negative public perception surrounding yawning is presumably a result of the temporal associations between reduced states of alertness and sleep/wake cycles^31,32^, i.e., yawns are known to occur when individuals are bored or drowsy so this act might indicate one’s disinterest or reduced mental status. Some researchers have therefore speculated that yawning serves a primary communicative role, in which yawns signal internal states to others^33,34^. However, a central signaling perspective fails to take into account that spontaneous yawns are widespread among solitary species, and often occur when alone even among gregarious animals^11,35^. Moreover, yawns are triggered by a multitude of factors, and are associated with a markedly variable array of contexts, stimuli and internal states (i.e., not just when we are bored or sleepy)^7,36^, and as a result could not serve as reliable signals. Nonetheless, the presence of others, i.e., audience or mere presence effects^37,38^, tends to diminish the expression of yawning. Early research has shown that spontaneous yawns are indeed less common among people in crowded environments^10^, as well as when being observed by a researcher in a laboratory^39^. It is unclear whether the reduced tendency to yawn in these settings is due to the negative signaling value of this response to others (i.e., a top-down mechanism), or from heightened arousal levels produced by these contexts that would act to naturally reduce yawning.

Similar socially-modulated effects have been observed for the expression of contagious yawning. The administration of intranasal oxytocin, a hormone and neuropeptide that enhances social perception and awareness^40^, has been shown to increase the likelihood that people actively inhibit and conceal their yawns, while also decreasing the tendency for any expressed yawns to be accompanied by overt cues of the response^41^. In comparison to those receiving a placebo saline solution, participants administered 30 IU of intranasal oxytocin were more likely to stifle their urge to yawn and required a greater length of exposure to a contagious stimulus prior to yawning. Moreover, among those that actually showed yawn contagion, individuals that received oxytocin were more likely to cover their mouth and clench their jaw during the act of yawning, and were less likely to display bodily stretching and produce auditory sighs during this response^41^.

In a more recent and targeted investigation of audience effects on yawn contagion, we explicitly examined how varied degrees of social presence in a laboratory setting altered contagious yawning^42^. Given the wide array of stimuli that elicit social presence effects, we tested how both implied (i.e., a “recording” webcam facing the participants) and actual social presence (i.e., a researcher in the testing room) influenced contagious yawning to video stimuli presented on a computer monitor. The results from this experiment showed that both implied and real social presence significantly diminished the self-reported expression of yawn contagion in comparison to control settings (e.g., a webcam that is off or a testing room that does not contain a researcher). Participants in the “recording” webcam or researcher present conditions also reported a greater urge to yawn without doing so when viewing the video stimuli^42^. Thus, the prospect, or actual presence, of others watching seems to inhibit even this inherently social form of yawning.

As an extension of this line of research, here we conducted five experiments to investigate the effects of real-world social presence on contagious yawning in virtual reality (VR). Although not without its limitations, utilizing VR in experimental psychology and the cognitive sciences offers numerous advantages^43,44^, providing greater levels of control and manipulation over traditional experimental procedures/settings. Furthermore, the capability of VR to potentially block out the external environment and engage the audience with its own stimuli allows for a level of immersion that typical displays cannot provide^45^. This is the first study to our knowledge which leverages VR to study the factors influencing yawning.

**Figure 1:**
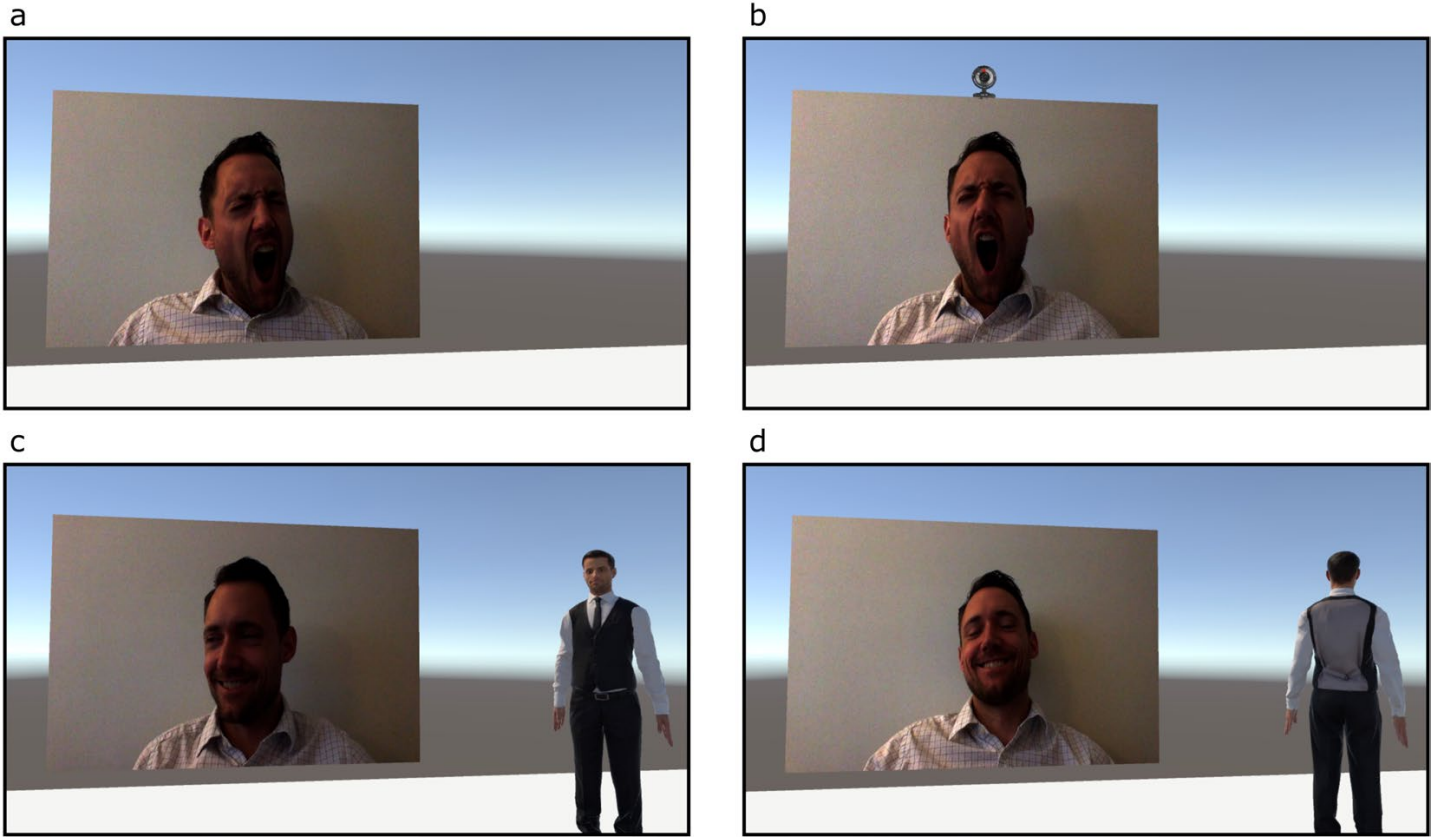
Depiction of varying VR stimuli for Experiments 1–4, including the presentation screen and social presence manipulations (not exhaustive). (**a**) Depicts the VR presentation for both conditions in Experiment 1; (**b**) depicts the high social presence condition for Experiment 2; (**c**) depicts high social presence condition for Experiment 4; (**d**) depicts the low social presence condition for Experiment 4.

**Figure 2:**
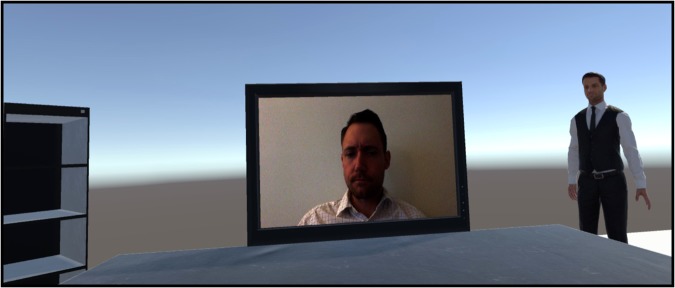
Depiction of the VR environment for Experiment 5. Designed to represent a typical laboratory experience, participants were instructed to sit down at the desk prior to the stimulus presentation.

We first assessed how actual social presence in the testing environment (i.e., a live researcher present) altered contagious yawning in VR, and then how both implied and actual forms of social presence *within* the virtual environment (i.e., embedding recording devices and humanoid avatars within the scene) modified this response (Fig. 1). In particular, the first experiment compared participant responses to contagious yawning stimuli presented in VR between conditions where a researcher either indicated that they would be sitting in the testing room during the virtual testing or not. The second and third experiments examined how the presence of an implicitly or explicitly recording webcam embedded within VR altered contagious yawn response rates within this setting. In the second experiment, the virtual webcam was either accompanied by a red light or not, while in the third experiment the researcher specifically instructed participants in advance that the webcam in the simulation would be recording their behavior during testing, or there was no mention of this device. The fourth experiment assessed how the presence of a motionless humanoid avatar within the scene influenced contagious yawning responses in VR. In particular, contagious yawning was compared between conditions when this figure was directly facing and looking towards the participants or was oriented 180 degrees in the opposite direction facing away. Lastly, the fifth experiment examined how contagious yawning was influenced by the presence of an onlooking humanoid avatar using a more realistic and representative virtual simulation. In particular, the humanoid avatar in this experiment swayed slightly and moved its limbs in a realistic way, mimicking natural behavior, and the virtual scene was designed to represent a more typical laboratory environment similar to our past research^42^ with a smaller presentation screen positioned on a desktop (Fig. 2). Thus, the design of this experiment allowed us to test the effect of avatar realism and display/room size and appearance. This final experiment also measured the immersive properties of the VR experience (including a sense of presence)^46^ to assess whether this varied between conditions and influenced yawning rates. In short, our operationalization of social presence both in the real world and in VR involved reproducing stimuli that are known to affect performance in actual reality (e.g., an active webcam, a person) in a VR environment. Note, however, that whether these stimuli will have the same effect in VR as in actual reality is very much an open question as the social presence of items in VR may require some level of psychological involvement and/or behavioral engagement^47^.

To the extent that placing a participant in a VR situation diminishes awareness or concern regarding the world beyond the VR setting, by inducing a mental state in which the user feels as though they are present within and can act upon the computer environment^48^, we made the following predictions based on the conditions of each experiment. For Experiment 1, unlike Gallup *et al*.^42^, we predicted that the presence of a researcher in the testing room would be equivalent to having no researcher in the room. In contrast, we predicted that placing a recording webcam in the VR environment (Experiment 2) or explicitly referring to the recording VR webcam (Experiment 3), would create an implied social presence and reduce the number of yawns and/or increase the urge to yawn without doing so. Similarly, we predicted that the actual presence of a humanoid avatar in the VR setting or having that person look at the participant (Experiments 4 and 5) would yield behaviors indicative of yawn inhibition, i.e., reduced yawn frequency and/or an increased urge to yawn.

## Results

### Experiment 1: Researcher Present/Absent

**Figure 3:**
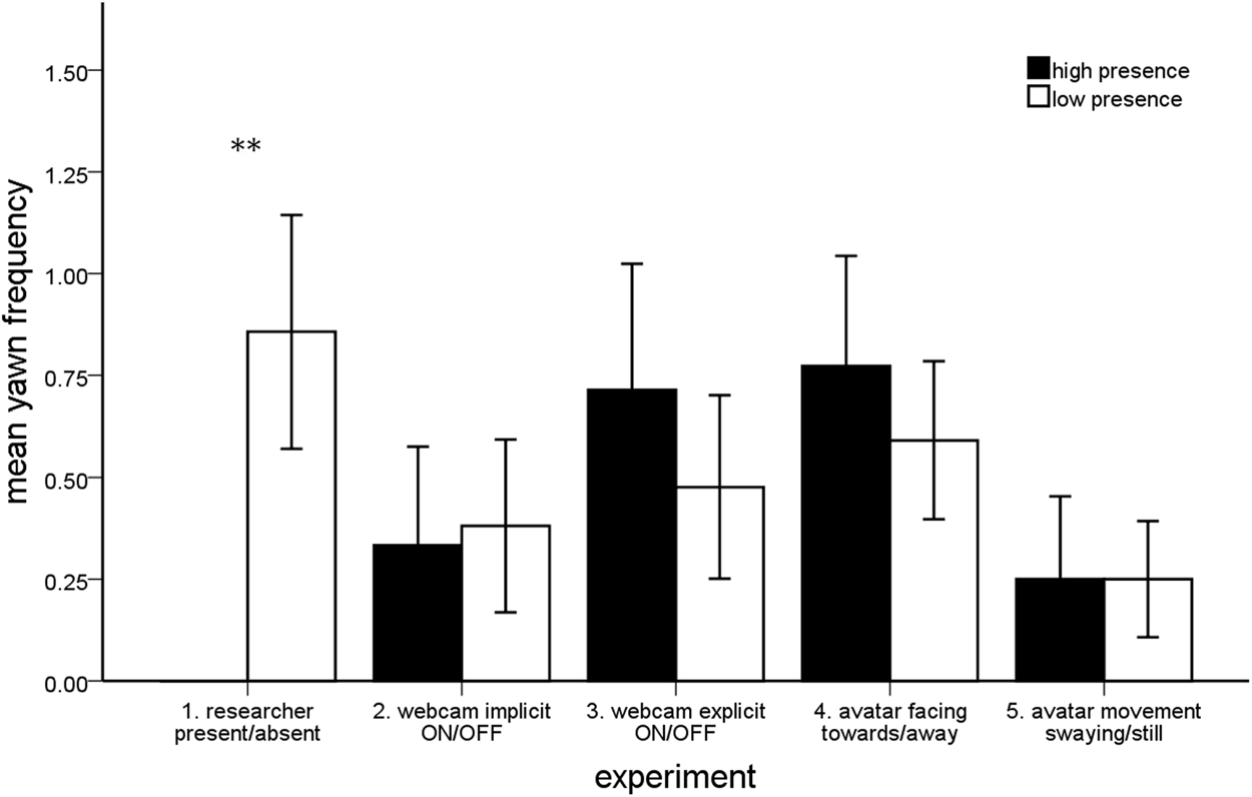
The mean number of contagious yawns per person across the high and low social presence conditions of all five experiments (SEM) (**p < 0.01).

**Figure 4:**
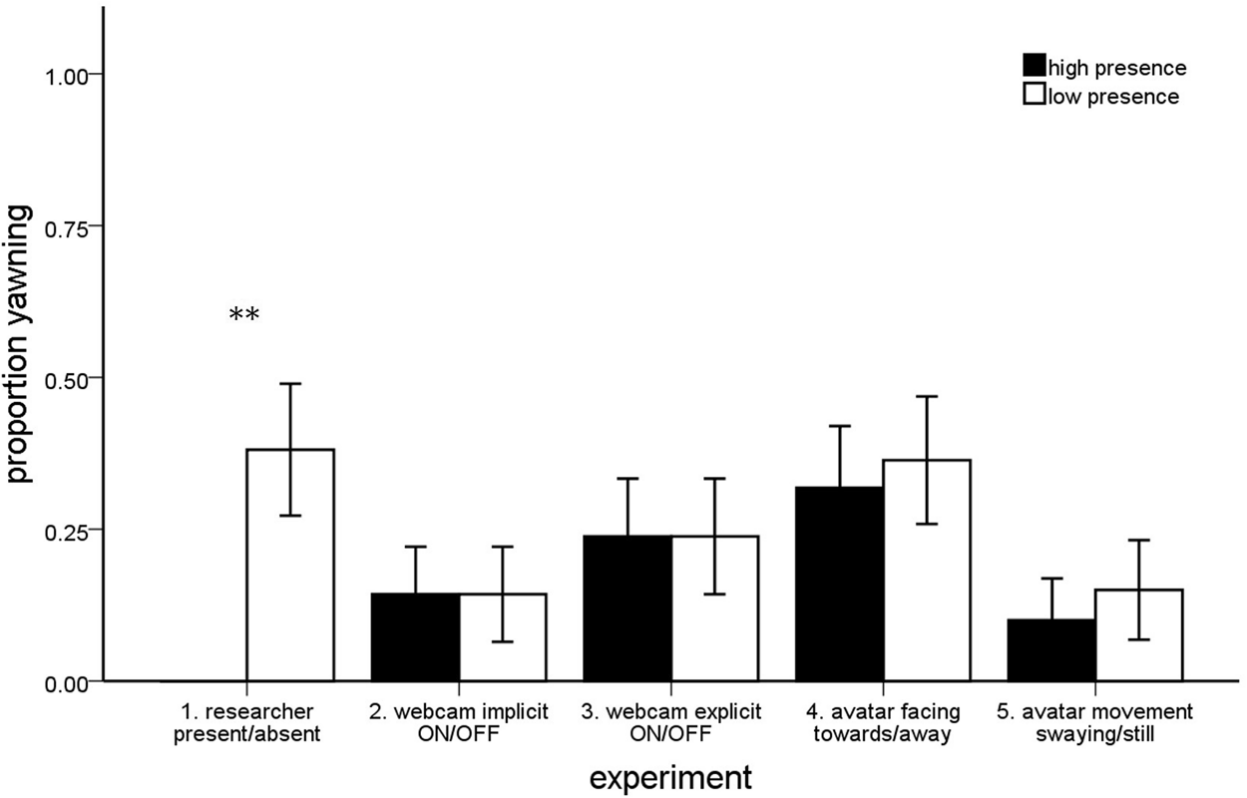
The proportion of participants that yawned contagiously across the high and low social presence conditions of all five experiments (SEM) (**p < 0.01).

A total of eight participants (38.1%) yawned during this experiment. There was a significant difference in the number of contagious yawns (*Z* = 2.546, *p* = 0.011; Fig. 3) and in the proportion of yawners (McNemar’s test, *p* = 0.008; Fig. 4) across social presence conditions. In particular, the presence of the researcher completely inhibited this response. In the Researcher Present (high social presence) condition not a single yawn was observed, while in the Researcher Absent (low social presence) condition participants yawned a total of 18 times (*M* ± SEM: 0.86 ± 0.29) in the simulated environment. However, there was no difference in the self-reported urge to yawn across conditions (McNemar’s test, *p* = 0.688).

### Experiment 2: Webcam Implicit ON/OFF

A total of four participants (19.0%) yawned during this experiment. There was no significant difference in the number of contagious yawns (*Z* = 0.184, *p* = 0.854; Fig. 3) or in the proportion of yawners (McNemar’s test, *p* = 1.000; Fig. 4) across the social presence conditions. Effectively no difference in contagious yawning was observed across conditions. In the Webcam ON (high social presence) condition participants yawned a total of seven times (0.33 ± 0.24), while in the Webcam OFF (low social presence) condition participants yawned for a total eight times (0.38 ± 0.21). There was also no difference in the self-reported urge to yawn across conditions (McNemar’s test, *p* = 0.125).

### Experiment 3: Webcam Explicit ON/OFF

A total of seven participants (33.3%) yawned during this experiment. Similar to Experiment 2, there was no significant difference in the number of contagious yawns (*Z* = 0.857, *p* = 0.391; Fig. 3) or in the proportion of yawners (McNemar’s test, *p* = 1.000; Fig. 4) across social presence conditions. In the Webcam ON (high social presence) condition participants yawned a total of 15 times (0.71 ± 0.31), while in the Webcam OFF (low social presence) condition participants yawned for a total 10 times (0.48 ± 0.22). Likewise, there was also no difference in the self-reported urge to yawn across conditions (McNemar’s test, *p* = 0.250).

### Experiment 4: Humanoid Avatar Facing Towards/Away

A total of ten participants (45.4%) yawned during this experiment. There was no significant difference in the number of contagious yawns (*Z* = 0.769, *p* = 0.442; Fig. 3) or in the proportion of yawners (McNemar’s test, *p* = 1.000; Fig. 4) across social presence conditions. In other words, the looking direction of the humanoid avatar had no influence on yawning behavior of participants in this experiment. In the Avatar Facing Towards (high social presence) condition a total of 17 yawns were observed (0.77 ± 0.27), while participants yawned a total 13 times in the Avatar Facing Away (low social presence) condition (0.59 ± 0.19). There was also no difference in the self-reported urge to yawn across conditions (McNemar’s test, *p* = 1.000).

### Experiment 5: Humanoid Avatar Movement Swaying/Still

A total of four participants (20.0%) yawned during this experiment. As predicted, there was a significant difference in the participants’ total immersion scores across the social presence conditions (*Z* = 2.187, *p* = 0.029), with participants reporting greater levels of immersion in the Avatar Movement Swaying condition (high social presence: 28.00 ± 1.17) compared to Avatar Movement Still condition (low social presence: 25.25 ± 1.38). However, there was absolutely no difference in the number of contagious yawns (*Z* = 0.000, *p* = 1.000; Fig. 3) or the proportion of yawners (McNemar’s test, *p* = 1.000; Fig. 4) across these two conditions. In both the Avatar Swaying (high social presence) and the Avatar Still (low social presence) conditions a total of five yawns were observed (0.25 ± 0.20; 0.25 ± 0.14). There was also no difference in the self-reported urge to yawn across conditions (McNemar’s test, *p* = 0.453).

Moreover, total immersion scores did not predict whether participants yawned in the Avatar Swaying (*Z* = 1.204, *p* = 0.229) or Avatar Still (*Z* = 0.106, *p* = 0.916) conditions. Similarly, this measure failed to predict the reported the urge to yawn in either condition (Avatar Swaying: *Z* = 1.591, *p* = 0.112; Avatar Still: *Z* = 0.306, *p* = 0.759). Although fewer yawns were observed in this more realistic VR setting when compared to Experiment 4, the difference in overall frequency and occurrence was not significant between the two conditions in which the avatar was facing front and still (i.e., the high social presence condition of Experiment 4 and the low social presence condition of Experiment 5: *Z* = 1.685, *p* = 0.092; Fisher’s exact test: *p* = 0.284). Thus, neither avatar realism nor display/room size and appearance impacted contagious yawning.

## Discussion

Conditions where others are around and potentially watching often changes the behavior of humans and non-human animals^37,38^. This report sought to extend previous research examining these so-called audience, or social presence, effects on the expression of contagious yawning in humans. By utilizing VR to study yawn contagion, we investigated the effects of both actual and simulated real-world social presence manipulations on this response through five separate experiments.

Based on previous research we had thought that placing participants within a VR environment would diminish their awareness of actual reality^48^, and with it, abolish our previous finding that a researcher in the testing room reduces the number of yawns people produce and increases the tendency that they fought off the urge to yawn^42^. The opposite result was obtained. Replicating, and extending our previous findings, we found that even when a person is placed in a VR environment, the real presence of a single researcher is sufficient to diminish the expression of contagious yawning in this setting. In other words, yawning behavior was affected by the live researcher presence even though participants were exposed to the virtual environment and unable to see this individual. In sum, Experiment 1 yielded an unexpected discovery that the presence of a researcher in the testing room significantly inhibits subsequent yawn contagion in VR, even though participants are occluded from the researcher and within a virtual environment when presented with the yawning stimuli.

One explanation for this unexpected outcome is that VR setup was not sufficient to shield participants from the real context and thus the social presence on the outside permeates their VR experience and alters socially contagious responses within. In light of the fact that Gallup *et al*.^42^ found that an *implied* social presence in the form of a live recording webcam is sufficient to inhibit yawning behaviors, we reasoned that a recording webcam placed within the participant’s virtual world would similarly serve to reduce the expression of yawn contagion. Surprisingly this was not what our investigations revealed. Participants in a virtual environment were immune to a webcam embedded in VR both when the recording state of the webcam was implicit (i.e., labeled with or without a red light) as in Experiment 2, or made explicit by the researcher prior to testing as in Experiment 3. Moreover, and even more surprising, Experiments 4 and 5 revealed that the actual presence of a humanoid avatar within the participant’s VR environment did not lead to a modulation of the contagious yawning response rates. This was true even when that person was directly facing the participants and displaying naturalistic movements, creating a more immersive experience within VR.

Collectively these data indicate that participants are quite sensitive to the social contagion of yawning within a virtual environment, which is consistent with recent VR research on other forms of contagious/reflexive behavior (i.e., gaze following)^49^. And yet, surprisingly, the participants were immune to the visual social signals of explicit (Experiments 4 and 5) and implied (Experiments 2 and 3) social presence displayed within this setting; but were extraordinarily sensitive to the unseen real presence of an experimenter outside of VR, i.e., in the testing room. The dynamics and social effects of virtual reality appear to be profoundly different from those in actual reality, with the latter superseding and dominating those of VR. These are evident both in the failure of varied real-world social presence effects within VR to have any significant impact on a social contagion trigger, which was true even for the more realistic VR simulations of Experiment 5, and by the effect of actual social presence outside the VR environment in Experiment 1 to alter social contagion to stimuli presented within this setting. Thus, within the domain of behavioral contagion, the social effects of VR are very unlike reality itself.

These findings suggest a major difference in the perception of social factors within and behind the scenes in VR, yet limitations to this study should be acknowledged. First, we suspect that the time spent in VR prior to testing and stimulus exposure is an important factor. As with our previous study^42^, it remains unknown whether participants would habituate to the researcher present condition over time and resume a more typical contagious yawning pattern thereafter. For example, past research has shown that social presence effects from wearing eye-trackers^50^ diminishes if participants wear these devices for a short period of time prior to experimentation^51^. Although results from Experiment 5 show no change in total immersion across the length of testing (from experimental block 1 to block 2), further research is needed to assess how any socially-mediated reduction in yawn contagion may dissipate over extended periods of time following various manipulations in VR. In addition, although the mere presence of an onlooking humanoid avatar was insufficient to alter yawning, it is possible that modifications to this figure could change the results. We attempted to construct a humanoid avatar that possessed realistic features in Experiment 4, and added naturalistic movement properties to this individual in Experiment 5, but further research could include even more advanced behavioral features to enhance avatar realism and interaction^44^. Such modifications could help avoid the “uncanny valley” of affinity towards entities of increasing but not complete human likeness^52^ and enhance the social presence, or sense of being with another via psychological involvement and/or behavioral engagement^47^, experienced from these conditions in VR. Related to these issues, it is possible that participants could be “present” and experience a sensation of being in a real place even more during the VR simulations. The results from Experiment 5 indicate that average presence scores were near the midpoint of the 7-point Likert scale used^53^. Thus, future research in this area could aim to improve place and plausibility illusions^54^ and/or suspensions of disbelief^55^ among participants within VR through modifications to the procedures and/or simulations. These issues notwithstanding, the current findings demonstrate the power of actual human observation on responses to social stimuli presented in VR, and provide a baseline for further work in this area.

It remains unclear whether the results in Experiment 1 are due to the enhanced recognition and social awareness of the negative perceptions of yawning when in the presence of others, or if this was a result of more bottom-up processes (i.e., heightened arousal) inhibiting the mechanisms that naturally trigger the primitive form of this response^11^. To date, the evidence for socially-mediated decreases in yawn contagion seem to be due at least in part to the former^41,42^. These factors could potentially be disentangled in the current setup by recording both gaze direction^50^ and physiological measures from participants exposed to social presence manipulations when engaged in VR^56^. It would be interesting to explore whether social perception or arousal factors have differential effects on the diminished expression of contagious versus spontaneous yawns. It seems plausible that since spontaneous yawns are triggered by internal neurophysiological processes^3^, the inhibition of this response within crowded environments or under direct observation^10,39^ is most likely a result of the latter.

Methodologically, this study provides a novel approach to the study and manipulation of contagious yawning. While previous comparative research has used computer animations to induce contagious yawning in chimpanzees^57^, this is the first study we know of to trigger this behavior within the context of a virtual environment. The rate of contagious yawning in VR without any social presence (i.e., low social presence condition in Experiment 1: 38.1%) was quite similar to yawning rates when using the same stimulus in traditional laboratory procedures^15,16^, and thus serves as a valid method for future research. Given the potential advantages of VR over traditional laboratory manipulations, refining this method in follow-up experiments could lead to a standardized approach to studying yawn contagion^58^. Further research could create humanoid avatars that yawn within the simulation, building from recent work examining human-robot interactions^59^. Previous studies have already begun to use VR in comparative research^60^, and thus similar methods could be applied to investigate yawn contagion in non-human animals.

Overall, this report adds to a growing literature showing that social presence can be a powerful deterrent of yawning in humans. In addition to improving upon methodological limitations to the current experiments, future studies should specifically examine the mechanism(s) contributing to the reduced yawning rate when in the presence of live onlookers. Given the potential adaptive function(s) and psychological correlates to yawning in humans, we believe the inhibition to yawning in some social settings deserves further investigation. Moreover, these experiments offer two insights into human behavior and cognition in VR. First, social presence in actual reality can have a profound impact on the perception of, and responses to, the virtual environment. Second, social factors influencing our behavior in the real world can have both similar (in the case of contagious yawning) and differing (in the case of social presence) effects when present in VR. Together, these findings could have broad and important applications for VR research across the psychological and cognitive sciences.

## Methods

Participants were recruited during the summer and fall of 2017 and the summer of 2018 through the Psychology Research Participation System at University of British Columbia (UBC) in Canada. All experiments were carried out in accordance with approved human ethics guidelines, and all participants provided informed consent prior to partaking in this study. The experimental protocols were approved by local ethics board, UBC’s Behavioral Research Ethics Board (H10-00527).

Upon entering the lab, participants were escorted to a testing room where they were seated at a table in front of a Schenker XMG P507 laptop computer running an HTC Vive Virtual Reality device. Participants were provided brief instructions on how to wear and use the VR headset and were instructed to pay close attention to a contagious yawning stimulus that would be presented within the simulation during testing. The stimulus presented in VR was the same as in our previous study^42^, which was initially developed by Platek *et al*.^15^ and has been used in other studies^16,41^. This stimulus consisted of a series of consecutive clips of people yawning, laughing and displaying neutral expressions (170 sec total viewing time; videos available from corresponding author upon request), and previous studies have shown that ~40% participants yawn following exposure to this stimulus in control conditions^15,41^. All clips were randomized, and were presented in 4:3 aspect ratio (details on screen distance and placement per experiment below). The experiment was designed for VR and executed using Unity (2017.4.1f1). The virtual room was calibrated so that participants could sit comfortably on a swivel chair facing the VR computer. Participants were given no explicit instructions regarding head movement, so they were free to move within the virtual environment. No sound accompanied the VR stimuli.

A repeated-measures design was used whereby participants sampled in each of the five experiments were exposed to two separate contagious yawning stimulus presentations in VR that varied in the degree of social presence (one high, one low; see below). The experimental block order was counterbalanced, and each presentation was separated by a 5-minute interval to avoid carryover effects of yawn contagion from one condition to the next^61,62^. Following exposure to each condition in the virtual environment, participants removed the Vive VR headset and filled out a brief paper questionnaire about their yawning behavior (see Supplemental Material)^42^. In addition, participants were covertly recorded by a webcam on the laptop running the Vive software in the testing room which provided an objective measurement of yawning. Subsequent scoring of >80% of these recordings by two independent researchers revealed very high inter-rater reliability for yawning behavior (yes/no: Cohen’s *K* = 0.847; frequency: intraclass correlation = 0.957). Given there is also substantial agreement between video-confirmed and self-report yawning under similar laboratory conditions^8,41^, questionnaire responses were used for participants when the webcam recordings were disrupted or participants requested their videos not be scored (14.8% of recordings). All yawns in the current study (N = 98) were considered contagious. Although concerns have been raised about the possibility of mislabeling some spontaneous yawns as contagious^63^, the former are very infrequent^10^ and predominantly clustered within the hours just prior to sleep and immediately after waking^31,32^. Across all five experiments, the testing of each condition only lasted 170 sec and was conducted outside these critical yawning periods. Furthermore, to date, spontaneous yawns have not been observed across any other VR experiments within this laboratory (A. Kingstone, personal communication).

### Analyses

Due to non-normal distributions of the yawning data across each of the five experiments, the number of contagious yawns was compared across social presence conditions using a Wilcoxon signed rank test. McNemar’s (chi square) tests were used to compare the proportion of yawners (binary) and those reporting the urge to yawn (binary) across social presence conditions. For Experiment 5, a Wilcoxon signed rank test was also used to compare the immersive properties between the two social presence conditions, while Mann-Whitney U tests were used to compare how these scores varied both between participants that yawned or not as well as those that reported the urge to yawn or not. Finally, contagious yawn frequency and occurrence was compared between similar conditions of Experiments 4 and 5 using Mann-Whitney U and Fisher’s exact tests. All analyses were performed in IBM SPSS v.24, with the alpha set to 0.05.

### Experiment 1: Researcher Present/Absent

This experiment was designed to investigate how contagious yawning to stimuli presented in VR was influenced by the presence of a researcher in the testing room. The VR room was created using Unity (2017.4.1) and was set up such that a 15 m by 10 m virtual screen playing the contagious yawning stimulus was approximately 20 meters away from the participant, subtending a visual angle of approximately 41.6° horizontal by 28.4° vertical. Participants sat on a chair in the middle of a white virtual floor surrounded by the default Unity skybox (see Fig. 1a). A total of 26 participants (23 female; *M* ± SD: 25.77 ± 10.48 yrs) were recruited for this study, and all testing occurred between 900 h and 1630 hr. Data were excluded from five participants because the VR computer crashed or did not run properly. Using a repeated-measures design, participants were each exposed to two separate conditions (order counterbalanced) while viewing the contagious yawning stimulus. In the Researcher Present (high social presence) condition, participants were informed just prior to fitting the Vive VR headset that the researcher (a 23-year-old Caucasian male) would be sitting within arm’s reach in the room with them during the VR experience (similar to Gallup *et al*.^42^). In the Researcher Absent (low social presence) condition, the same researcher indicated that he would be leaving the room during testing. There were no significant differences in the age or sex ratio of the participants, hours of sleep the night before, or testing times between experimental block orders.

### Experiment 2: Webcam Implicit ON/OFF

This experiment was designed to investigate how contagious yawning was influenced by the presence of an implicitly recording webcam (see Fig. 1b). The virtual environment was the same as in Experiment 1, with the exception of the additional virtual webcam placed just above the presentation screen. The webcam subtended an approximately 4.2° × 4.2° visual angle. A total of 22 participants (13 female; 22.64 ± 5.23 yrs) were recruited for this study, and all testing occurred between 1030 h and 1330 h. Data were excluded from one participant because the VR computer crashed. Using a repeated-measures design, participants were exposed to two separate conditions (order counterbalanced) while viewing the contagious yawning stimulus. In the Webcam ON (high social presence) condition, the webcam had a bright red light on it, while in the Webcam OFF (low social presence) condition no light was present. The researcher indicated that they would be leaving the room during testing for both conditions. There were no significant differences in the age or sex ratio of the participants, hours of sleep the night before, or testing times between experimental block orders.

### Experiment 3: Webcam Explicit ON/OFF

This experiment was designed to investigate how contagious yawning was influenced by the presence of an explicitly recording webcam positioned just above the presentation screen in VR. The virtual environment was the same as in Experiment 2, with the exception of a red light placed on the top edge of the virtual webcam. A total of 21 participants (17 female; 19.95 ± 1.47 yrs) were recruited for this study, and all testing occurred between 1000 h and 1430 h. Using a repeated-measures design, participants were exposed to two separate conditions (order counterbalanced) while viewing the contagious yawning stimulus. In the Webcam ON (high social presence) condition, participants were informed just prior to fitting the Vive device that the webcam above the stimulus would be recording their behavior during the simulation (although no light was present as in Experiment 2). In the Webcam OFF (low social presence) condition, there was no mentioning of the webcam prior to testing (matching the OFF condition in Experiment 2). There were no significant differences in the age or sex ratio of the participants, hours of sleep the night before, or testing times between experimental block orders.

### Experiment 4: Humanoid Avatar Facing Towards/Away

This experiment investigated how contagious yawning to stimuli presented in VR was influenced by the presence of an onlooking, but motionless, humanoid avatar within the same simulated environment depicted in Experiments 1–3. The avatar was 0.70 m wide (with arms slightly extended at the side) and 1.86 m tall, subtending a visual angle of approximately 10.1° horizontal by 26.7° vertical. A total of 22 participants (17 female; 20.59 ± 1.89 yrs) were recruited for this study, and all testing occurred between 1000 h and 1630 h. Using a repeated-measures design, participants were exposed to two separate conditions (order counterbalanced). In the Avatar Facing Towards (high social presence) condition, a humanoid avatar was positioned to the right of the stimulus presentation screen looking directly at the participant during testing (see Fig. 1c). In the Avatar Facing Away (low social presence) condition, this figure was in the same location but oriented 180 degrees to face in the opposite direction (see Fig. 1d). There were no significant differences in the age or sex ratio of the participants, hours of sleep the night before, or testing times between experimental block orders.

### Experiment 5: Humanoid Avatar Movement Swaying/Still

This experiment was designed to investigate how contagious yawning was influenced by the presence of an onlooking humanoid avatar within a more realistic and representative virtual simulation. In particular, the virtual scene was designed to represent a more typical laboratory environment similar to our past research^42^ with a smaller presentation screen positioned on a desktop (Fig. 2). The humanoid avatar in this experiment (which was the same figure used in Experiment 4) was also designed to be more realistic and human-like, displaying naturalistic movement. The 1 m × 0.7 m screen was located on a virtual desk in front of the participant at a distance of 1.8 m, subtending a visual angle of approximately 30.6° horizontal by 20.8° vertical. The participants sat on a swivel chair 4 m away from the avatar, now subtending a visual angle of approximately 9.8° horizontal by 25.9° vertical. The virtual shelf opposite the avatar was 3.7 m away from the participant, subtending a visual angle of 12.0° horizontal by 25.3° vertical. For this experiment we had participants complete a previously validated questionnaire^53^ to assess how the immersive properties of the VR experience varied between conditions and whether it predicted contagious yawning within this context. Total immersion was calculated by taking the composite of four questions designed to measure presence and three questions designed to measure flow (each on a 7-point Likert scale; see Georgiou & Kyza)^53^. A total of 22 participants (15 female; 19.91 ± 1.63 yrs) were recruited for this study, and all testing occurred between 1030 h and 1430 h. Data were excluded from one participant because the VR computer crashed or did not run properly and from one other because they did not look at the stimulus during testing. Using a repeated-measures design, participants were exposed to two separate conditions (order counterbalanced). In the Avatar Movement Swaying (high social presence) condition, the humanoid avatar was positioned to the right of the stimulus presentation screen swaying slightly and displaying naturalistic limb movements while looking directly at the participant during testing (video demonstration in Supplemental Material). In the Avatar Movement Still (low social presence) condition, this figure was in the same location and orientation but remained completely motionless as in Experiment 4. There were no significant differences in the age or sex ratio of the participants, hours of sleep the night before, or testing times between experimental block orders.

## Electronic supplementary material

VR Video Demo

Supplementary Information

## Data Availability

The datasets generated during and/or analyzed during the current study are available from the corresponding author on reasonable request.
