# Supplementary material for: Contagious yawning in virtual reality is affected by actual, but not simulated, social presence
Source: Sci Rep. 2019 Jan 22;9:294. doi: 10.1038/s41598-018-36570-2 (PMC6342947; doi:10.1038/s41598-018-36570-2)
Supplement: Supplementary file 2 — Supplementary Information [file 41598_2018_36570_MOESM2_ESM.docx]

**Supplemental Materials for the following article:**

**Contagious yawning in virtual reality is affected by actual, but not simulated, social presence**

Andrew C. Gallup^1*^, Daniil Vasilyev^2^, Nicola Anderson^2^, and Alan Kingstone^2^

^1^Psychology Program, SUNY Polytechnic Institute, Utica, NY 13502 USA

^2^Department of Psychology, University of British Columbia, Vancouver, BC Canada V6T 1Z

^*^Correspondence: [a.c.gallup@gmail.com](mailto:a.c.gallup@gmail.com)

**Questionnaire**

Date: ________________

Time: ________________

Sex: Male Female

1. Did you yawn during the course of the experiment? Yes No

2. If yes, how many times did you yawn? ________________

3. Did you have the urge to yawn during the experiment? Yes No

4. How many hours did you sleep last night? ________________

5. How old are you? ________________

---------------------To be filled out by the researcher---------------------

Condition: ________________
